# Serum fetuin-a and risk of thoracic aortic aneurysms: a two-sample mendelian randomization study

**DOI:** 10.3389/fendo.2024.1361416

**Published:** 2024-02-26

**Authors:** Yang Chen, Jiayi Zhu, Xin Guo, Chenghui Cao, Xuan Xiao, Botao Zhu, Shuwei Weng, Die Hu, Yonghong Luo, Shuai Wang, Sha Wu, Jia He, Yang Yang, Daoquan Peng

**Affiliations:** ^1^ Department of Cardiovascular Medicine, The Second Xiangya Hospital, Central South University, Changsha, China; ^2^ Research Institute of Blood Lipid and Atherosclerosis, Central South University, Changsha, China

**Keywords:** fetuin-A, thoracic aortic aneurysm, thoracic aortic diameter, mendelian randomization analysis, mechanisms

## Abstract

**Background:**

Recent studies have revealed a significant decrease in serum fetuin-A levels in atherosclerotic aneurysms, indicating that fetuin-A may play a protective role in the progression of arterial calcification. However, the specific mechanism behind this phenomenon remains unclear. We aimed to examine the association between fetuin-A levels in thoracic aortic aneurysms (TAAs) and risk of TAAs and to evaluate whether this association was causal.

**Methods:**

A total of 26 SNPs were selected as instrumental variables for fetuin-A in 9,055 participants of European ancestry from the CHARGE consortium, and their effects on thoracic aortic aneurysm and decreased descending thoracic aortic diameter were separately estimated in 353,049 and 39,688 individuals from FinnGen consortium. We used two-sample Mendelian randomization (MR) analysis to examine the causal association. At the same time, we employed various methods, including random-effects inverse variance weighting, weighted median, MR Egger regression, and MR PRESSO, to ensure the robustness of causal effects. We assessed heterogeneity using Cochran’s Q value and examined horizontal pleiotropy through MR Egger regression and retention analysis.

**Results:**

Fetuin-A level was associated with a significantly decreasing risk of thoracic aortic aneurysm (odds ratio (OR) 0.64, 95% CI 0.47 - 0.87, *P* = 0.0044). Genetically predicted fetuin-A was also correlated with the decreased descending thoracic aortic diameter (β = -0.086, standard error (SE) 0.036, *P* = 0.017).

**Conclusions:**

Serum fetuin-A level was negatively associated with risk of TTAs and correlated with the decreased descending thoracic aortic diameter. Mendelian randomization provides support for the potential causal relationship between fetuin-A and thoracic aortic aneurysm.

## 1 Introduction

Aortic aneurysm is a critical cardiovascular disease characterized by exceptionally high mortality rates. It is a complex multifactorial condition influenced by both genetic and environmental risk factors. Thoracic aortic aneurysms (TAAs) refer to the dilation of the ascending part of the aorta, accounting for one-third of the incidence of aortic aneurysm, which may lead to dissection or aortic rupture (1). It is related to age, male, smoking, hypertension, family history and genetic susceptibility. Atherosclerosis is an infrequent underlying cause in most cases. The precise etiology, however, remains incompletely elucidated, underscoring the practical significance of early biomarker identification and detection.

Fetuin-A, also referred to as α2-Heremans-Schmid glycoprotein or α2-HS glycoprotein, is a heterodimeric plasma glycoprotein composed of 282 amino acids in the A chain and 27 amino acids in the B chain (2). It is primarily secreted by the liver and can also be found widely expressed in various tissues (3). Fetuin-A has also been shown to play a vital role in the development of several disorders. It plays a role in regulating calcium metabolism, osteogenesis, and insulin signaling pathways. Additionally, it functions as a heterotopic calcification inhibitor, protease inhibitor, inflammatory mediator, anti-inflammatory mediator, and atherogenic factor (4). Recent studies (5, 6) have demonstrated that elevated levels of plasma fetuin-A are linked to an elevated risk of myocardial infarction and stroke. This suggests that fetuin-A plays a role in the pathophysiology of cardiovascular diseases. In an observational study (7) comprising 30 cases of atherosclerotic aortic aneurysms, 15 cases of Marfan syndrome, 30 cases of peripheral arterial diseases, and healthy controls, Szeberin et al. discovered a significant lower in the serum level of fetuin-A in the atherosclerotic aortic aneurysm group. This finding lends support to the notion that fetuin-A plays a protective role in the process of arterial calcification. The plasma samples from six patients with TAA before and after surgery, as well as from six healthy controls, were analyzed by Kazamia et al. (8). The findings revealed a significant decrease in the concentration of alpha-2-HS glycoprotein (AHSG), also known as fetuin-A, in preoperative plasma samples from TAA patients compared to healthy controls. These results suggest that AHSG (fetuin-A) holds promise as a potential biomarker for TAA. However, the involvement of fetuiu-A in the occurrence and progression of TAAs remains elusive.

Mendelian Randomization (MR) is a method that utilizes genetic variations, particularly single nucleotide polymorphisms (SNPs), as instrumental variables (IVs) to establish causal relationships between diseases (outcomes) and risk factors (exposures) (9). To date, no Mendelian Randomization (MR) analysis has assessed the association between fetuin-A and thoracic aortic aneurysm. Nevertheless, clinical data indicates a significant reduction in serum fetuin-A levels within the aortic aneurysm group. Hence, it is imperative to further investigate the potential causal link between fetuin-A levels and thoracic aortic aneurysm.

Our hypothesis posits that serum fetuin-A levels are inversely correlated with the risk of thoracic aortic aneurysms. Given the limited evidence regarding the causal relationship between fetuin-A and the risk of thoracic aortic aneurysm, our objective is to employ Genome-Wide Single Nucleotide Polymorphism Array(GWAS) data to examine the causal between serum fetuin-A levels and the risk of thoracic aortic aneurysms. Additionally, we will explore potential causal associations through MR analysis, utilizing two distinct datasets sourced from GWSA data.

## 2 Methods

### 2.1 Study population and instrumental variables selection

The summary-level variants associated with fetuin-A expression were obtained from the CHARGE Consortium, which included 9,055 participants of European ancestry (10). This study further derived 26 SNPs fetuin-A using a criterion of P < 5 × 10^−8^ and LD r^2^ < 0.4. The F-statistic indicates the strength of the relationship between SNPs and exposures. A higher F-statistic, typically greater than 10, suggests a lower likelihood of causing weak instrument bias. The genetic information of selected SNPs in detail is shown in **Supplementary Table 1**.

The summary datasets of thoracic aortic aneurysms were obtained from FinnGen release 9. We chose genetic association data from a total of 353,049 European participants. Descending thoracic aortic diameter were derived from Pirruccello et al., which trained deep learning model to identify genetic variants associated with thoracic aortic diameter in 39,688 individuals (11). The maximum size in the elliptical minor axis during the cardiac cycle was defined as the aortic diameter, and its association with genetic variants was analyzed in UK biobank patients. All MR Analyses satisfy three basic assumptions: 1) instrumental variables are strongly correlated with exposure factors; 2) Instrumental variables are not correlated with confounding factors; 3) Instrumental variables are not directly related to the results, and their impact on the results can only be reflected through exposure.

### 2.2 Mendelian randomization analysis

We conducted MR analysis using the ‘TwoSampleMR’ package in R. The inverse variance weighted (IVW) meta-analysis method was chosen as the primary MR analysis, as it provides optimum accuracy with decent IV quality (12). Different supplementary methods, namely MR-Egger, weighted median, simple mode, weighted mode, and maximum likelihood were utilized to evaluate the causal association. The intercept of the MR-Egger regression can represent horizontal pleiotropy, and it can be applied in the presence of unbalanced pleiotropy (13). The weighted median method continues to offer a consistent estimate of the causal effect, even when over 50% of the instrumental variables are deemed invalid (14). The robust adjusted profile score (RAPS) method can better address the bias caused by potential weak instruments and pleiotropy.

### 2.3 Sensitivity analyses

To further ensure IV quality, extensive sensitivity analyses were performed. We used Cochran’s Q test to evaluate heterogeneity between each IV. MR-Egger regression was employed to assess pleiotropy, with an MR-Egger intercept indicating its existence. MR pleiotropy residual sum and outlier (MR-PRESSO) method is also a powerful complement for testing pleiotropy in MR, with the global test detecting horizontal pleiotropy, and the outlier test can correct the estimate by removing outliers if necessary (15). Furthermore, we utilized the PhenoScanner database (http://www.phenoscanner.medschl.cam.ac.uk/) to evaluate the potential associations between the selected IVs and any confounding factors that could potentially impact outcomes (16). A leave-one-out analysis was conducted to identify potentially influential SNPs with significant effects on remaining IVW results.

### 2.4 Statistical analysis

All analyses were performed using R software (version 4.1.2). The ‘MRPRESSO’ package (version 1.0) was used for the MR-PRESSO method, and ‘mr.raps’ package (version 0.2) for the RAPS method. An online web tool (https://sb452.shinyapps.io/power/) was utilized for power analysis. A *P* < 0.05 was considered statistically significant in the primary analyses and Sensitivity analyses.

## 3 Results

### 3.1 The causal effect of fetuin-A level on thoracic aorta outcomes

**Figure 1:**
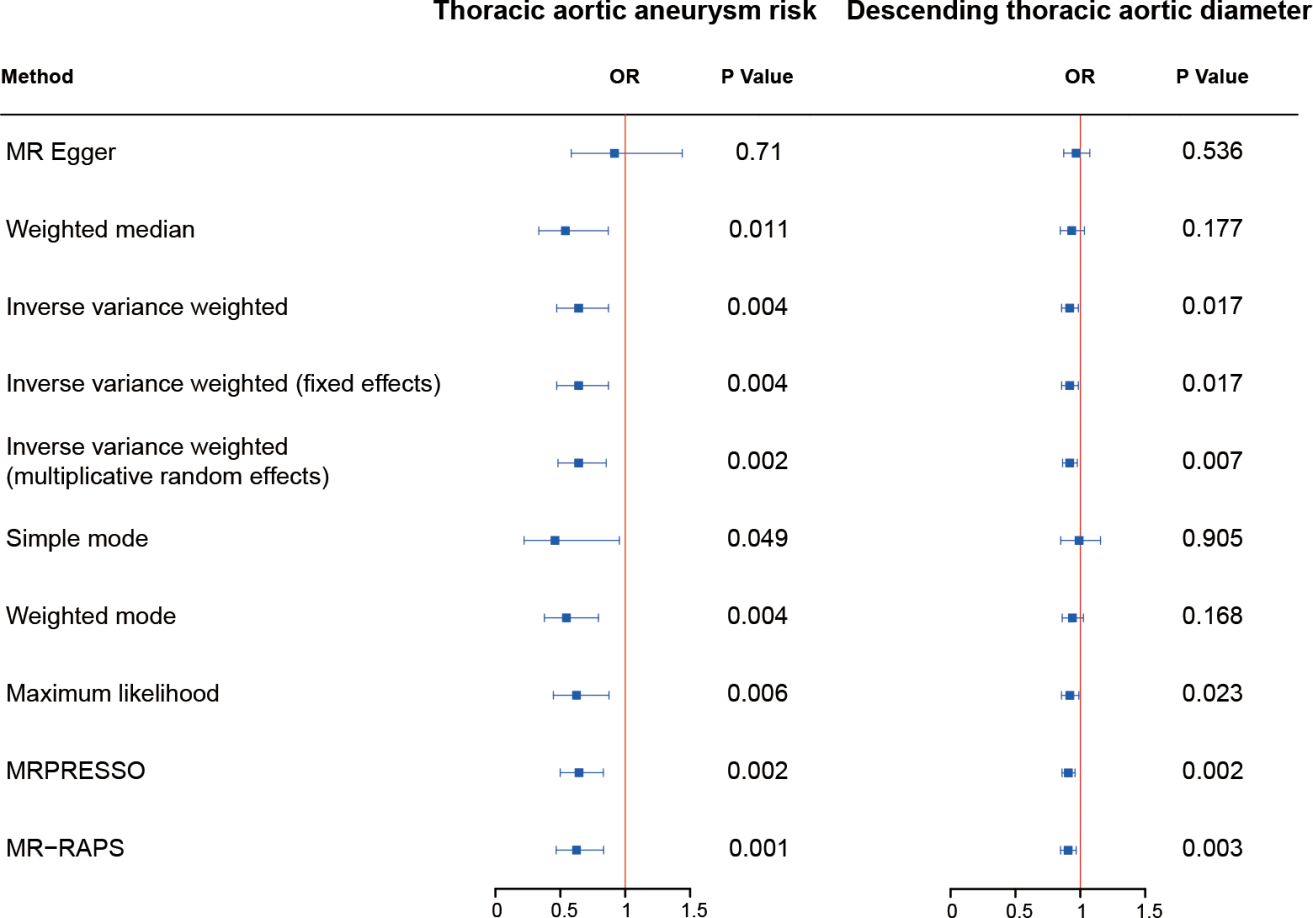
The causal effect of fetuin-A level on thoracic aorta outcomes. The odds ratios (ORs) are adjusted to genetically predict serum fetuin-A levels for assessing the risk of thoracic aortic aneurysm. OR, odds ratio.

**Figure 2:**
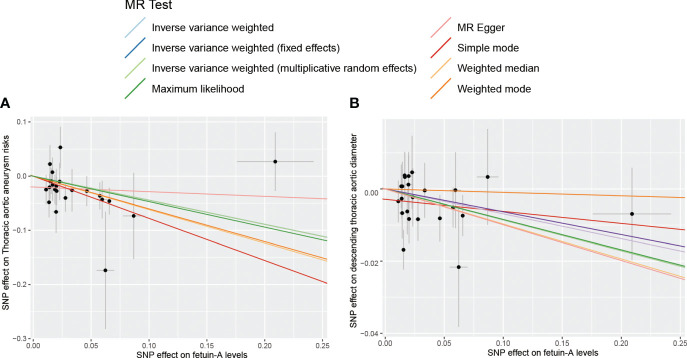
The scatter plot for MR analyses of fetuin-A levels and the risk of thoracic aortic aneurysm using different MR methods. **(A)** The effect of SNPs on the risk of thoracic aortic aneurysm; **(B)** The effect of SNPs on the risk of descending thoracic aortic diameter.

Complete MR results are shown in the **Supplementary Table 1**. Among four pollution exposures, IVW estimates that fetuin-A expression was associated with a significantly decreasing risk of thoracic aortic aneurysm (OR = 0.64, 95% CI: 0.47 - 0.87, *P* = 0.0044). Genetically predicted fetuin-A was also correlated with the decreased descending thoracic aortic diameter (β = -0.086, SE = 0.036, *P* = 0.017) (**Figure 1**, **Table 1**). The ORs in all seven models (fixed effects IVW, multiplicative random effects IVW, MR-Egger, weighted median, simple mode, weighted mode, and maximum likelihood) demonstrated the same direction, as evidenced in the negative slopes of all the lines between fetuin-A levels and aortic outcomes (**Figure 2**). The direction and significance of MR-PRESSO and RAPS analyses are consistent with IVW for both thoracic aortic aneurysm risks (MR-PRESSO: OR = 0.64, 95% CI: 0.50 - 0.83, *P* = 0.0024; RAPS: OR = 0.62, 95% CI: 0.47 - 0.83, *P* = 0.0014) and descending thoracic aortic diameter (MR-PRESSO: β = -0.097, SE = 0.028, *P* = 0.0021; RAPS: β = -0.099, SE = 0.034, *P* = 0.0033).

**Table 1:** Causal effects assessed by IVW, RAPS, and MR-PRESSO, and sensitivity analyses for fetuin-A level on aortic outcomes.

Outcome	Methods	Beta	SE	P-value	MR-Egger’s P-value	Cochran Q test’s P-value	MR-PRESSO Global Test P-value
Thoracic aortic aneurysm	IVW	-0.44	0.15	0.0044*	0.046^#^	0.631	0.537
	RAPS	-0.47	0.15	0.001*			
	MR-PRESSO	-0.44	0.13	0.002*			
Descending thoracic aortic diameter	IVW	-0.09	0.04	0.017*	0.192	0.765	0.814
	RAPS	-0.10	0.03	0.003*			
	MR-PRESSO	-0.10	0.03	0.002*			

*P-value < 0.05 for mendelian randomization analysis.

^#^P-value < 0.05 for sensitivity analysis.

IVW, inverse variance weighted. RAPS, robust adjusted profile score. MR-PRESSO, mendelian randomization pleiotropy residual sum and outlier.

### 3.2 Sensitivity analysis

The Cochran’s Q test detected no heterogeneity in our study. MR-Egger regression indicated the existence of pleiotropy in the relationship between fetuin-A level and the risk of thoracic aortic aneurysm (*P* = 0.046). However, no pleiotropy was detected using the MR-PRESSO global tests and no SNP was removed. The leave-one-out analysis also determined that no IV substantially influenced overall MR analysis results for all significant exposure-outcome estimates (**Supplementary Figure 1**). All SNPs passed the correlation strength threshold, as the minimum F-statistic was 33.18, indicating unlikely weak instrument bias for MR analysis (**Supplementary Table 2**). The PhenoScanner search results revealed that no IVs were associated with confounding traits of thoracic aortic aneurysm risk or descending thoracic aortic diameter (**Supplementary Table 3**). The MR results in our study were relatively robust, indicating that high fetuin-A level is a protective factor for thoracic aortic aneurysm and enlargement of the descending thoracic aorta.

## 4 Discussion

This study employed a bidirectional two-sample Mendelian randomization (MR) design to investigate the associations between fetuin-A and thoracic aortic aneurysm, marking the first instance of such analysis. Two-sample Mendelian randomization analyses demonstrated that higher fetuin-A levels were associated with a significantly reduced risk of thoracic aortic aneurysm (odds ratio (OR) = 0.64, 95% confidence interval (CI): 0.47 - 0.87, *P* = 0.0044) and a decrease in the diameter of thoracic aortic aneurysms (β = -0.086, standard error (SE) = 0.036, *P* = 0.017).

The presence of aortic aneurysms pose a significant risk to cardiovascular health and can potentially be life-threatening. It can be categorized into two types: thoracic aortic aneurysm and abdominal aortic aneurysm, each with distinct underlying causes. The development of thoracic aortic aneurysm primarily revolves around changes in the extracellular matrix (17). As research progresses, an increasing body of evidence has confirmed the correlation between calcification of aneurysm walls and the mortality rate associated with aneurysms (18, 19).

Kazamia et al (8). utilized liquid chromatography-tandem mass spectrometry to examine proteins extracted from 14 thoracic aortic aneurysms (TAAs) tissue samples and 12 non-aneurysmal thoracic aortic tissue samples. They also analyzed plasma samples from 6 TAA patients before and after surgery and 6 healthy control individuals. The findings revealed a notable reduction in the concentration of Alpha-2-HS-glycoprotein (AHSG) (Also known as fetuin-A) in the preoperative plasma samples compared to those from the healthy controls, indicating that AHSG might serve as a promising biomarker for TAA. This study represented the first observation of the relationship between Fetuin-A and patients with aortic aneurysms of various causes. Szeberin et al (7). conducted a single-center cross-sectional observational study involving 105 patients, including 30 with atherosclerotic aortic aneurysm, 15 with Marfan syndrome, 30 with peripheral arterial disease, and 30 healthy controls. They analyzed the serum levels of fetuin-A. The findings revealed a significant reduction in serum fetuin-A levels in the atherosclerotic aortic aneurysm group, supporting the notion that fetuin-A plays a protective role in the development of arterial calcification. These results further substantiate the hypothesis that fetuin-A may serve as an inhibitor of arterial calcification in atherosclerotic patients, independent of diabetes and in the absence of obvious renal dysfunction. This discovery has the potential to enhance diagnostic capabilities for this condition. This is the initial proposition suggesting that fetuin-A plays a role in the development of thoracic aortic aneurysms (TAAs) and may potentially offer a protective effect.

Currently, the potential connection between fetuin-A and TAAs remains uncertain. We employed a two-sample Mendelian randomization study to explore whether a causal link exists between them, and to assess the causal association between fetuin-A and aortic diameter. The findings demonstrated a significant correlation between fetuin-A expression and a reduced risk of thoracic aortic aneurysms (OR=0.64, 95% CI: 0.47-0.87, *P*=0.00044). Additionally, gene-predicted fetuin-A was also linked to a reduction in the diameter of thoracic aortic aneurysms (β = -0.086, SE = 0.036, *P* = 0.017).

Previous studies have indicated that the size of thoracic aortic aneurysms is a critical predictor of rupture. Aneurysms with a diameter of 5 to 6 centimeters tend to grow more rapidly and are at a higher risk of rupturing compared to smaller aneurysms (20). In recent years, an increasing number of studies have demonstrated that aortic aneurysms often coincide with vascular calcification. The calcification of arteries reduces their elasticity, which worsens the occurrence and progression of aneurysms (21). As is widely known, the primary pathophysiological mechanisms of thoracic aortic aneurysms (TAAs) involve inflammation, apoptosis of smooth muscle cells, and the secretion of proteases, which result in the degradation of the extracellular matrix (ECM). Matrix metalloproteinases (MMPs), particularly MMP-2 and MMP-9, are activated and increased in expression in thoracic aortic aneurysm (TAA) (22, 23). The action of MMPs, which damage ECM, can result in a weakened medial layer of the aorta, potentially leading to an aneurysm (24). Our findings demonstrate a significant association between the level of fetuin-A and a reduced risk of thoracic aortic aneurysm, along with a decrease in the diameter of the thoracic aortic aneurysms. Meanwhile, fetuin-A, acting as an inhibitor of ectopic calcification and proteases, is suggested to play a protective role in the development of arterial calcification, according to the relevant literature. Consequently, we propose that the mechanism by which fetuin-A protects against thoracic aortic aneurysms appears to involve the inhibition of arterial calcification, an influence on arterial diameter, and the suppression of MMPs.

Nonetheless, our study has several limitations that warrant consideration. Firstly, future updated Mendelian randomization analyses using summary statistics from larger genetic studies should be conducted to confirm the findings of our current MR study. A significant constraint in our study is the inability to directly assess the associations of individual genetic variants with potential confounding factors related to the link between fetuin-A levels and TAA. This limitation stems from our lack of knowledge regarding potential confounders and the unavailability of individual-level data. Notably, potential confounding variables, such as disease type, disease state, age, sex, and others, may introduce biases into the causal inferences drawn. A more comprehensive exploration of the functions of selected instrumental variables and the refinement of algorithms within the MR framework could potentially help mitigate the impact of confounding factors. Thirdly, the measured fetuin-A level at a specific timepoint may be influenced by transient factors, such as age and inflammation, which might not accurately represent the lifelong fetuin-A levels determined by the encoding gene. Fourth, it is worth noting that the GWAS data used primarily comprised samples of European ancestry, which may limit the generalizability of our findings to other racial or ethnic populations. Finally, although our study suggests a causal association between CSF fetuin-A levels and TAA, it’s important to recognize that mendelian randomization analysis provides a predictive result that requires further validation. Therefore, the establishment of a causal relationship and the underlying pathological mechanisms must be explored. Although clinical trials have suggested the prioritization of fetuin-A detection in thoracic aortic aneurysms, further evidence-based confirmation is still required through large-scale clinical randomized controlled trials.

## 5 Conclusion

In conclusion, Mendelian randomization analysis has revealed a negative correlation between serum fetuin-A levels and the risk of TAA, accompanied by a lower in the diameter of TAA. The findings of our study provide persuasive evidence for the early detection and diagnosis of thoracic aortic aneurysms. Nevertheless, the underlying mechanism remains unclear at present and warrants further validation through animal experiments and large-scale clinical trials.

## Data availability statement

The original contributions presented in the study are included in the article/**Supplementary Material**. Further inquiries can be directed to the corresponding author.

## Ethics statement

The present study exclusively utilized published or publicly accessible data. Ethical approval for each included study can be found in the primary publications, which also encompassed informed consent from all participants.

## Author contributions

YC: Investigation, Methodology, Validation, Writing – original draft, Writing – review & editing. DP: Funding acquisition, Project administration, Supervision, Writing – review & editing. JZ: Investigation, Methodology, Validation, Writing – review & editing. XG: Investigation, Methodology, Validation, Writing – review & editing. CC: Investigation, Methodology, Validation, Writing – review & editing. XX: Investigation, Methodology, Validation, Writing – review & editing. BZ: Investigation, Methodology, Validation, Writing – review & editing. SW: Investigation, Methodology, Validation, Writing – review & editing. DH: Conceptualization, Investigation, Methodology, Software, Writing – review & editing. YL: Conceptualization, Investigation, Methodology, Software, Writing – review & editing. SW: Conceptualization, Investigation, Methodology, Software, Writing – review & editing. SW: Conceptualization, Investigation, Methodology, Software, Writing – review & editing. JH: Conceptualization, Investigation, Methodology, Software, Writing – review & editing. YY: Conceptualization, Investigation, Methodology, Software, Writing – review & editing.

## Supplementary material

Supplementary Figure 1 The leave-one-out analysis plot and funnel plot of fetuin-A levels and the risk of thoracic aortic aneurysm. **(A)** Leave-one-out plot of fetuin-A and thoracic aortic aneurysm; **(B)** Leave-one-out plot of fetuin-A and thoracic aortic diameter; **(C)** Funnel plot of fetuin-A and thoracic aortic aneurysm; **(D)** Funnel plot of fetuin-A and thoracic aortic diameter.
