# Supplementary figures and images for: Serum fetuin-a and risk of thoracic aortic aneurysms: a two-sample mendelian randomization study
Source: Front Endocrinol (Lausanne). 2024 Feb 26;15:1361416. doi: 10.3389/fendo.2024.1361416 (PMC10925661; doi:10.3389/fendo.2024.1361416)

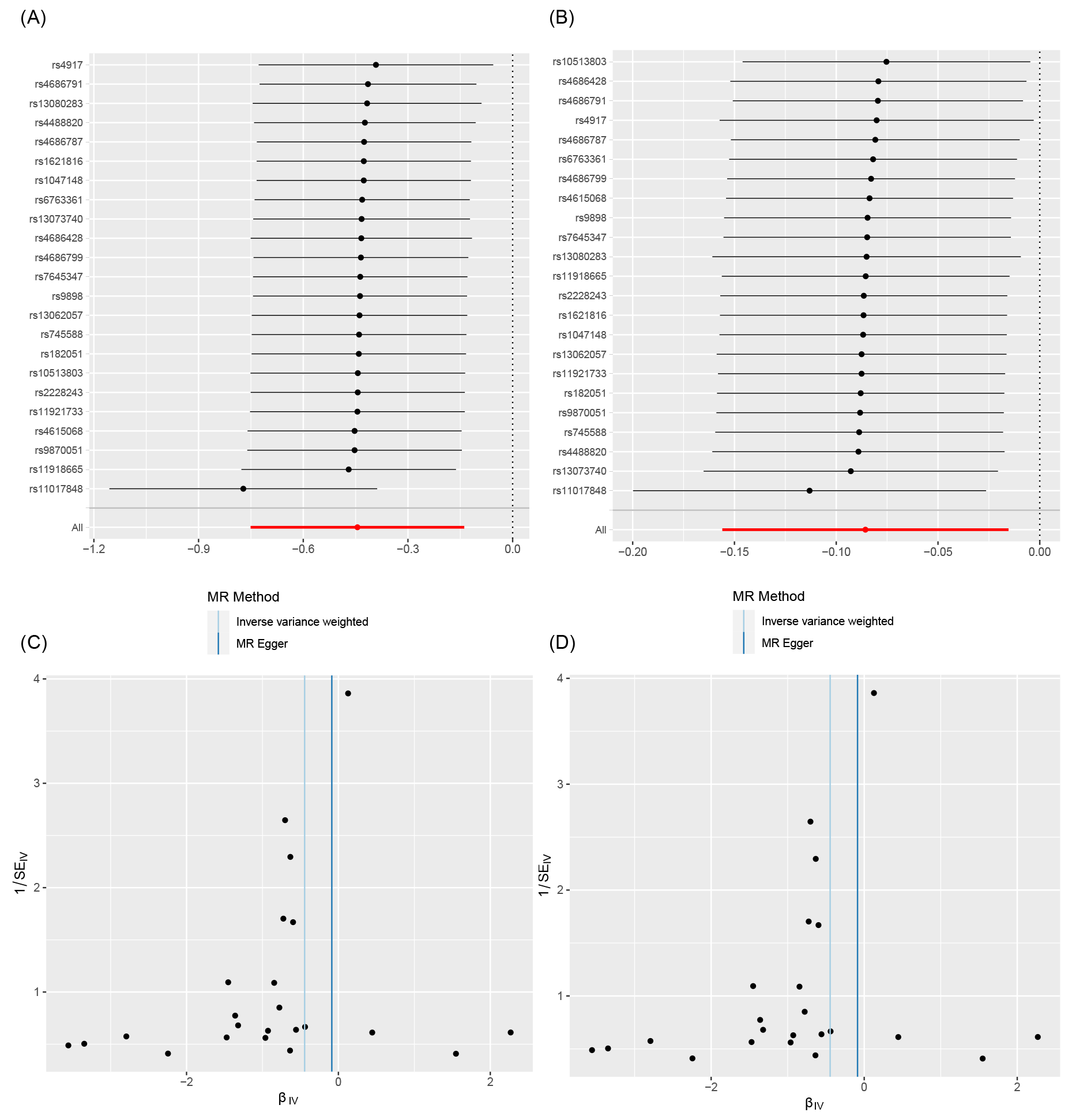

Supplement: Supplementary Figure 1 — The leave-one-out analysis plot and funnel plot of fetuin-A levels and the risk of thoracic aortic aneurysm. (A) Leave-one-out plot of fetuin-A and thoracic aortic aneurysm; (B) Leave-one-out plot of fetuin-A and thoracic aortic diameter; (C) Funnel plot of fetuin-A and thoracic aortic aneurysm; (D) Funnel plot of fetuin-A and thoracic aortic diameter. [file Image_1.tif]
