# Supplementary material for: Serum fetuin-a and risk of thoracic aortic aneurysms: a two-sample mendelian randomization study
Source: Front Endocrinol (Lausanne). 2024 Feb 26;15:1361416. doi: 10.3389/fendo.2024.1361416 (PMC10925661; doi:10.3389/fendo.2024.1361416)
Supplement: Supplementary file 2 [file Table_1.docx]

**Supplementary Table 1 Complete mendelian randomization estimates between fetuin-A level on aortic outcomes.** IVW, inverse variance weighted; FE-IVW, fixed effects inverse variance weighted; MRE-IVW, multiplicative random effects inverse variance weighted.

| Method | Thoracic aortic aneurysm risk | | |  | Descending thoracic aortic diameter | | |  |
| --- | --- | --- | --- | --- | --- | --- | --- | --- |
|  | Beta | SE | P |  | Beta | SE | P |  |
| MR Egger | -0.09 | 0.23 | 0.710 |  | -0.03 | 0.05 | 0.536 |  |
| Weighted median | -0.62 | 0.24 | 0.011* |  | -0.07 | 0.05 | 0.148 |  |
| IVW | -0.44 | 0.16 | 0.004* |  | -0.09 | 0.04 | 0.017* |  |
| FE-IVW | -0.44 | 0.16 | 0.004* |  | -0.09 | 0.04 | 0.017* |  |
| MRE-IVW | -0.44 | 0.15 | 0.002* |  | -0.09 | 0.03 | 0.007* |  |
| Simple mode | -0.78 | 0.37 | 0.049* |  | -0.01 | 0.08 | 0.904 |  |
| Weighted mode | -0.60 | 0.19 | 0.004* |  | -0.06 | 0.04 | 0.169 |  |
| Maximum likelihood | -0.47 | 0.17 | 0.006* |  | -0.08 | 0.04 | 0.023* |  |
| RAPS | -0.47 | 0.15 | 0.001* |  | -0.10 | 0.03 | 0.003* |  |
| MR-REPSSO | -0.44 | 0.13 | 0.002* |  | -0.10 | 0.03 | 0.002* |  |

* *P*-value < 0.05 for mendelian randomization analysis
